# Non-coding RNAs from seminal plasma extracellular vesicles and success of live birth among couples undergoing fertility treatment

**DOI:** 10.3389/fcell.2023.1174211

**Published:** 2023-06-22

**Authors:** Oladele A. Oluwayiose, Emily Houle, Brian W. Whitcomb, Alexander Suvorov, Tayyab Rahil, Cynthia K. Sites, Stephen A. Krawetz, Pablo E. Visconti, J. Richard Pilsner

**Affiliations:** ^1^ C.S. Mott Center for Human Growth and Development, Department of Obstetrics and Gynecology, School of Medicine, Wayne State University, Detroit, MI, United States; ^2^ Department of Biostatistics and Epidemiology, School of Public Health and Health Sciences, University of Massachusetts Amherst, Amherst, MA, United States; ^3^ Department of Environmental Health Sciences, School of Public Health and Health Sciences, University of Massachusetts Amherst, Amherst, MA, United States; ^4^ Division of Reproductive Endocrinology and Infertility, Department of Obstetrics and Gynecology, Springfield, MA, United States; ^5^ Center for Molecular Medicine and Genetics, Wayne State School of Medicine, Detroit, MI, United States; ^6^ Department of Veterinary and Animal Sciences, University of Massachusetts Amherst, Amherst, MA, United States; ^7^ Institute of Environmental Health Sciences, Wayne State University, Detroit, MI, United States

**Keywords:** seminal plasma, extracellular vesicles, small non-coding RNA, live birth, infertility, art

## Abstract

**Background:** Infertility remains a global health problem with male-factor infertility accounting for around 50% of cases. Understanding the molecular markers for the male contribution of live birth success has been limited. Here, we evaluated the expression levels of seminal plasma extracellular vesicle (spEV) non-coding RNAs (ncRNAs) in men of couples in relation with those with and without a successful live birth after infertility treatment.

**Method:** Sperm-free spEV small RNA profiles were generated from 91 semen samples collected from male participants of couples undergoing assisted reproductive technology (ART) treatment. Couples were classified into two groups based on successful live birth (yes, *n* = 28) and (no, *n* = 63). Mapping of reads to human transcriptomes followed the order: miRNA > tRNA > piRNA > rRNA> “other” RNA > circRNA > lncRNA. Differential expression analysis of biotype-specific normalized read counts between groups were assessed using EdgeR (FDR<0.05).

**Result:** We found a total of 12 differentially expressed spEV ncRNAs which included 10 circRNAs and two piRNAs between the live birth groups. Most (*n* = 8) of the identified circRNAs were downregulated in the no live birth group and targeted genes related to ontology terms such as negative reproductive system and head development, tissue morphogenesis, embryo development ending in birth or egg hatching, and vesicle-mediated transport. The differentially upregulated piRNAs overlapped with genomic regions including coding *PID1* genes previously known to play a role in mitochondrion morphogenesis, signal transduction and cellular proliferation.

**Conclusion:** This study identified novel ncRNAs profiles of spEVs differentiating men of couples with and without live birth and emphasizes the role of the male partner for ART success.

## Introduction

Infertility is the inability to conceive within 1 year of regular unprotected sexual intercourse. It remains a global reproductive problem affecting 15% of heterosexual couples and nearly 190 million individuals with the desire to conceive (Rutstein and Shah, 2004; Mascarenhas et al., 2012). The diagnosis of couple infertility involves the evaluation of both male and female factors (Smith et al., 2003; Wright et al., 2003; Campbell et al., 2021). However, approximately 30% of cases are unexplained with unknown etiology when the results of standard infertility evaluations are normal (Quaas and Dokras, 2008).

Due to increased access and delayed parenthood (Kocourkova et al., 2014; van Loendersloot et al., 2014), there is a steady rise in the use of ART including *in vitro* fertilization (IVF) or intracytoplasmic sperm injection (ICSI) (Sunderam et al., 2017). However, despite that ART has existed for several decades, the global overall success rate, as measured by live birth outcomes following a single embryo transfer, is approximately 30% (Smith et al., 2015; Gleicher et al., 2019; Khalife et al., 2020). This may prompt multiple attempts, constituting physical, financial and/or emotional burden among couples (Domar et al., 1992). Thus, understanding the molecular markers that are associated with live birth outcomes prior to the initiation of the ART treatment cycle may provide a pathway to enhance clinical care.

As such, profiling seminal plasma to identify novel markers of reproductive success has been proposed (Crawford et al., 2015; Ayaz et al., 2021; Rodriguez-Martinez et al., 2021). Seminal plasma is enriched in biologically active molecules including coding and non-coding RNAs (Vojtech et al., 2014; Ayaz et al., 2021; Rodriguez-Martinez et al., 2021; Oluwayiose et al., 2022), which are either found freely or encapsulated in extracellular vesicles (EVs). Importantly, there is a growing interest in unraveling the reproductive functions of ncRNAs retained in EVs found in seminal plasma. Findings suggest that injection of ncRNA cargo of seminal plasma EV (spEV) can modulate gene expression, potentially regulating biological processes involved in embryonic developments (Chen et al., 2016; Chen et al., 2021).

Recent findings from our group (Oluwayiose et al., 2022) complement prior evidence of ncRNA enrichment in spEVs (Vojtech et al., 2014; Joshi et al., 2022) and showed these ncRNAs were differentially expressed in men with poor semen quality, suggesting the relevance of spEV ncRNAs in reproductive outcomes. However, whether these spEV ncRNAs also serve as markers of live birth outcomes, the major goal of infertility treatment in clinical setting, is unknown. Thus, this study evaluated the expression of spEV ncRNAs in men of couples undergoing ART treatment in relation to those with and without live birth.

## Methods

*Study population.* The study population includes men (*n* = 91) of couples receiving fertility treatment at Baystate Medical Center located in Springfield, Massachusetts who agreed to participate in the Sperm Environmental Epigenetics and Developments Study (SEEDS). SEEDS is prospective observational cohort study designed to investigate the associations of male preconception endocrine disrupting chemical exposure with sperm epigenetics and subsequent early-life development. The inclusion criteria were male partners of at least 18 years old without vasectomy and fresh ejaculate sperm used for ART treatment while females were included if 40 years or younger. Relevant demographics (race, age, height, and weight), lifestyle factors (current and past alcohol and cigarette use), and medical history (diagnoses of infertility) data were collected by clinic personnel during the ART cycle for both partners. Male fertility diagnoses were based on semen quality parameters according to World Health Organization (WHO) reference values (Cooper et al., 2010) while female diagnoses were based on CDC criteria including polycystic ovarian syndrome, anovulation, tubal factors, diminished ovarian reserve and endometriosis (Wright et al., 2003). Females received ovarian stimulation protocols based on infertility diagnoses and medical history. Egg retrieval was carried out about 36 h after the leading follicle reached 18–20 mm diameter Written informed consent was obtained from eligible males who showed voluntary interest in participating in the study by the attending physicians. The study is approved by the institutional review boards at Baystate Medical Center and at the University of Massachusetts Amherst (IRB number: BH-12-190).

*Isolation of EVs from seminal plasma.* Following a 2–3 days abstinence period, freshly collected ejaculates were processed using a two-step (40% and 80%) gradient fractionation method (Wu et al., 2015) and the resulting seminal plasma was stored in −80°C until subsequent analysis. To isolate EVs and total RNA, frozen seminal plasma samples were thawed for 20 min at room temperature, followed by extraction of EV RNA isolation using Qiagen exoRNAeasy Midi Kit (Cat. #77144) with slight modification to manufacturer’s protocol (Enderle et al., 2015). Briefly, equal volume of PBS and seminal plasma were mixed, centrifuged at 12,000 g for 45 min at 4°C to pellet sperm and other debris, and the resulting supernatant carefully separated and pre-filtered through a 0.45 μm syringe (Whatman PURADISC 25, Cat.#6780-2505). This was mixed with one volume of DNA binding buffer (XBP) and centrifuged at 500 *g* for 3 min in a spin column. The column-bound EVs were then purified using XWP buffer and lysed with QIAzol prior to phase-separation with chloroform for total RNA isolation. The aqueous phase containing RNA was mixed with 2 volumes of 100% ethanol and transferred onto a spin column. The column-bound RNA was purified, washed in 80% ethanol and eluted in nuclease free water following by total RNA quantification and quality assessment using nanodrop and fragment analyzer respectively.

*Small RNA library preparation and sequencing.* End repair of the isolated total RNA was performed using T4 Polynucleotide kinase (NEB, Cat #M0201) according to manufacturer’s instructions. All steps including polyadenylated RNA library preparation, reverse transcription cDNA synthesis, and 6 cycles of PCR amplification followed modified SMARTer smRNA-Seq protocol (Clontech). SPRI beads were used for library cleanup, and size selection and quality assessment were performed using AMPure X beads and fragment analyzer, respectively. Multiplexed samples were loaded onto the flow cell using Illumina barcodes (1—24) and sequencing of 50-bp single-end reads was performed on an Illumina HiSeq 4000. Libraries construction and sequencing were conducted at the UMass Medical Genomics Core Facility.

*small RNA sequencing analysis.* The quality of the raw reads was assessed using FastQC (Andrews, 2010). Trimming of the first three nucleotides and polyA sequences as well as filtering of reads with low quality (PHRED score < 20) and reads shorter than 16 nt was performed using cutadapt (Martin, 2011). Sequencing contaminants were eliminated by alignment to UniVec database using Bowtie2 with default parameters (Langmead and Salzberg, 2012). Sequential alignment to human transcriptome databases for ncRNA biotypes (Rozowsky et al., 2019; Oluwayiose et al., 2022) followed the order: miRNA > piRNA > tRNA > rRNA>“other ncRNAs”>circRNA > lncRNA using STAR (v2.7.6a) (Dobin et al., 2013). Multi-mapping reads were assigned based on the best mapping quality. We allowed incremental mismatch: no mismatches in the reads ≤25 bases and 1 mismatch in 26–50 bases. Samtools (v1.9) (Li et al., 2009) was used for BAM/SAM data manipulation and counting of mapped reads. All raw and processed sequences were deposited into the GEO database (http://www.ncbi.nlm.nih.gov/geo/) under accession number GSE203494. Details of the count output were reported in our previous publication (Oluwayiose et al., 2022).

*Data analysis.* Biotype-specific counts were normalized using weighted trimmed mean of M-values TMM (Robinson and Oshlack, 2010) and species with normalized count ≤1 RPM in all samples were filtered. Male participants fertility diagnosis was defined based on WHO 5th centile reference cutoff points (Cooper et al., 2010) and were categorized based on couple live birth status (Yes/No, referent = Yes). Live birth was defined as clinically confirmed delivery of a living infant from the current ART treatment cycle. Differential expression analysis between men with and without successful live birth was performed using EdgeR (Robinson et al., 2010). ncRNA species with absolute value of log2 fold change (FC) ≥ 0.585 and FDR<0.05 was considered significant. Student’s *t*-test or the nonparametric *Mann–Whitney U* test was used to compare participants’ demographic and clinical characteristics between groups while proportions were compared using chi-square statistics (*p* < 0.05). All analyses were performed in R (v4.1.0) software.

*Gene target determination and functional annotation.* BEDTools (Quinlan and Hall, 2010) was first used to annotate the differentially expressed piRNAs to non-repeat genomic region obtained from piRBase and total human genome (https://www.gencodegenes.org/human/release_38lift37.html). Based on evidence suggesting shared gene-regulating mechanisms between piRNA and miRNA (Robine et al., 2009; Gainetdinov et al., 2022; Kamenova et al., 2022), we utilized miranda algorithm (Enright et al., 2003) to predict potential interactions between our differentially expressed piRNAs and 3′UTR mRNA using alignment score >160 and ∆G ≤ −10 kcal/mol. For differentially expressed circRNA, top 5% of predicted circRNA-miRNA pairs curated either in miranda or targetscan database were identified (Zhou et al., 2020). This was followed by the identification of conserved miRNA-gene pairs which were predicted in both miranda and targetscan. Ontology and pathway analyses of target genes were performed using metascape (https://metascape.org/gp/index.html#/main/step1) (Zhou et al., 2019).

## Results

The overall demographic and clinical characteristics of the participants (*n* = 91) are presented in Table1. The participants were mostly non-Hispanic white (81.3%) with an average age (years ±SD) and BMI (kg/m^2^) of 36.2 ± 6.0 and 29.9 ± 5.5, respectively. Table 1 also compares the demographic and semen characteristics of participants stratified based on couple live birth status (Live birth *n* = 28; no live birth, *n* = 63). There was no statistical difference (*p* > 0.05) in the couple demographics, infertility diagnoses and ART outcomes such as the number of oocytes retrieved and fertilized eggs between the two groups. As expected, the total counts of day 3 high quality embryos were significantly higher (*p* < 0.05) in the group with livebirth relative to the group without successful livebirth. While similar observation was also observed in day 5 high quality embryos, it did not achieve statistical significance (*p* > 0.05).

**TABLE 1:** Demographic and clinical features of SEEDS participants (*n* = 91) and stratification by live birth status.

**Couple demographics characteristics**				
Features	All participants (*n* = 91)	Live birth (*n* = 28)	No live birth (*n* = 63)	*p*-value^†^
	Mean ± SD/count (%)	Mean ± SD/count (%)	Mean ± SD/count (%)	
Male age (years)	36.2 ± 6.0	35.2 ± 5.2	34.6 ± 6.4	0.30
Male BMI (kg/m^2^)^a^	29.9 ± 5.5	29.5 ± 5.2	30.1 ± 5.7	0.67
Male race^b^				
Hispanic white	74 (81.3)	24 (88.9)	50 (87.7)	1.00
Others	10 (10.4)	3 (11.1)	7 (7.7)	
Female age (years)	34.0 ± 3.9	33.5 ± 3.8	34.2 ± 3.9	0.40
Female BMI (kg/m^2^)	27.3 ± 6.7	28.8 ± 8 .0	26.7 ± 6.0	0.20
Female race^c^				
Hispanic white	56 (61.5)	17 (94.4)	39 (90.7)	1.00
Others	5 (5.5)	1 (5.6)	4 (9.3)	

Couple infertility diagnoses				
	Median (Range)/count (%)	Median (Range)/count (%)	Median (Range)	
Sperm concentration(10^6^/mL)^d^	65.3 (4.2—667.5)	70.2 (6.6—667.5)	62.0 (4.2—442.5)	0.74
Total motility (%)	62.0 (12.0—91.0)	65 (33.0—88.0)	61.0 (12.0—91.0)	0.16
Normal morphology(%)^e^	7.3 (0.5—21.0)	7.0 (0.5—21.0)	6.5 (0.5—19.5)	0.86
Count(10^6^/ejaculate)^f^	162.5 (8.8—1170.0)	179.3 (8.6—876.0)	157.0 (13.1—1170.0)	0.80
Semen volume(mL)	3.0 (0.4—7.5)	2.7 (0.5—7.5)	3.1 (0.4—6.5)	0.42
Polycystic ovariansyndrome	16 (17.6)	5 (17.9)	11 (17.5)	1.00
Anovulation	7 (7.7)	3 (10.7)	4 (6.3)	0.77
Tubal factors	17 (18.7)	3 (10.7)	14 (22.2)	0.31
Diminishedovarian reserve	19 (20.9)	4 (14.3)	15 (23.8)	0.45
Endometriosis	10 (11.0)	3 (10.7)	7 (11.1)	1.00

Couple reproductive outcomes				
	Mean ± SD/count (%)	Mean ± SD/count (%)	Mean ± SD/count (%)	
Oocytes retrieved	13.9 ± 8.0	13.5 ± 5.9	14.0 ± 8.8	0.70
Oocytes fertilized	8.4 ± 6.2	8.7 ± 4.7	8.3 ± 6.7	0.75
High quality embryo				
Day3	75 (82.0)	27 (96.4)	48 (76.2)	0.04
Day5	34 (37.4)	14 (50.0)	20 (31.7)	0.15

Missing data:

^a^ Male BMI (*n* = 4).

^b^ Male race (*n* = 7).

^c^ Race female (*n* = 30).

^d^ Concentration (*n* = 1).

^e^ Normal morphology (*n* = 3).

^f^ Count (*n* = 1);.

^†^ Represents statistical comparison (Student’s t-test/*Mann–Whitney U* test/chi-square) of parameters between men and women with live birth (*n* = 28), and men and women without live birth (*n* = 63). Live birth was defined as successful birth from the current ART, cycle.

**FIGURE 1:**
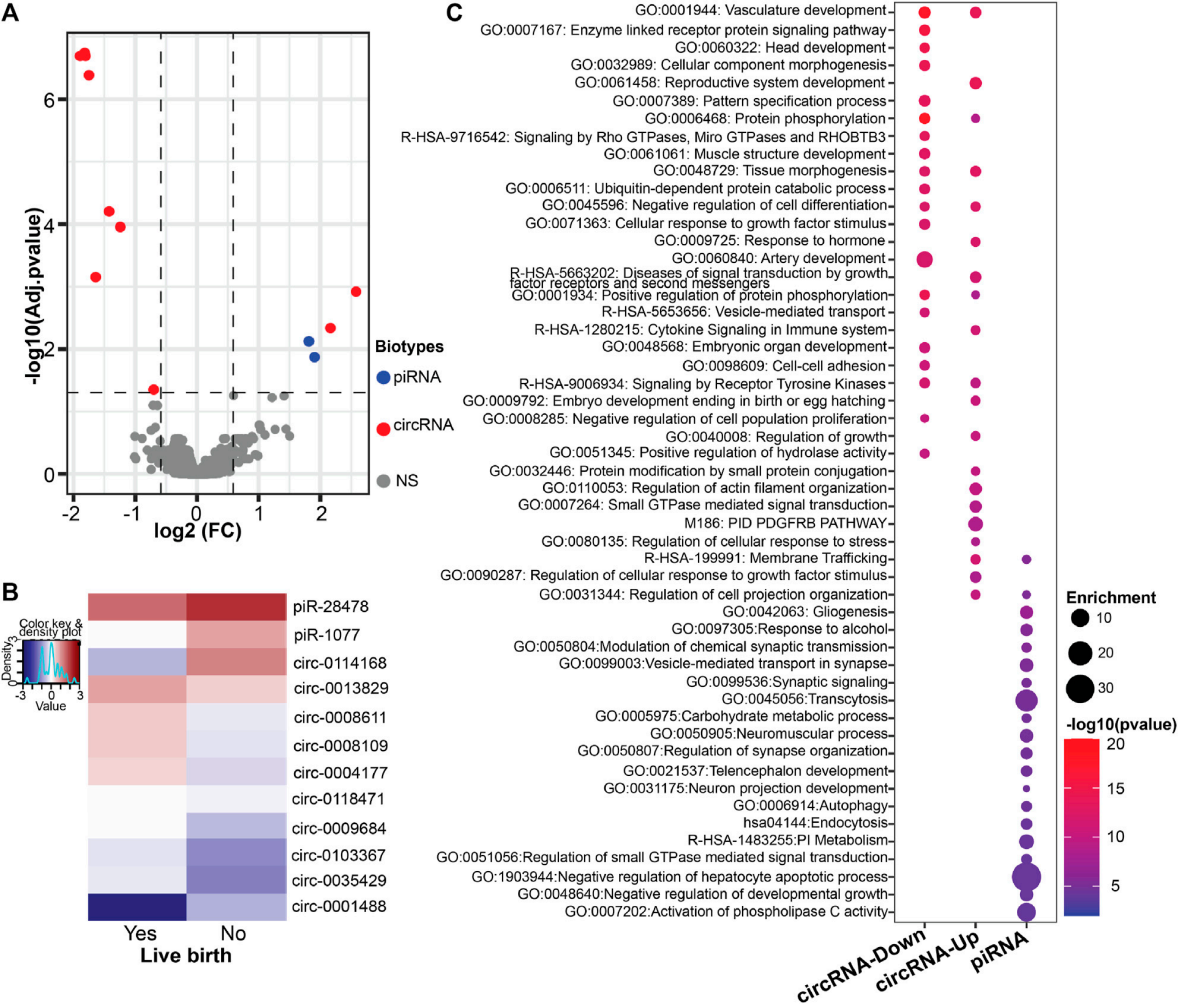
spEV ncRNA expression levels by live birth status (Yes/No, referent = Yes). **(A)** Volcano plot of differential expression of all identified spEV ncRNA biotypes. Dashed lines represent cutoff points [horizontal line = FDR of 0.05; vertical lines = absolute value of log2 fold change (FC) ≥ 0.585]. Colored and grayed datapoints are statistically significant and non-significant (NS) differentially expressed ncRNAs, respectively. **(B)** Heatmaps of all differentially expressed spEV ncRNAs **(C)** Dotplot of ontology analysis of differentially expressed spEV circRNA and piRNA gene targets.

First, we sought to identify spEV derived differentially expressed ncRNAs in men without successful live births compared to men with live births. We found a total of 12 ncRNAs were differentially expressed in men without live birth (Figures 1A, B; Supplementary Table S1). The most prominent differentially expressed ncRNA species were annotated to circRNAs (*n* = 10, 83%), of which the majority (80%) had lower expression in spEVs among men of couples without successful live birth compared to those with live birth. The remaining two differentially expressed spEV ncRNAs were annotated as piRNAs (piR-28478 and piR-1077), in which both were over-expressed in men without live birth compared to men of couples with a successful live birth.

Next, we performed gene ontology analyses to understand the biological significance of the differentially expressed circRNAs and piRNAs (Figure 1C). Potential gene target analyses revealed that the 10 downregulated circRNAs were associated with 69 miRNAs, which in turn, targeted 815 unique genes (Supplementary Tables S2, S3). The downregulated differentially expressed circRNAs gene targets were enriched in several developmental related ontology terms including head development, cell component morphogenesis, pattern specification process, muscle structure and artery development, vesicle-mediated transport and embryonic organ development. For the two upregulated circRNAs, 60 miRNAs were identified as potential target which in-turn resulted in 731 unique gene targets (Supplementary Tables S2, S3). Similar to those of the downregulated circRNAs, gene targets of the upregulated circRNAs showed enrichment in biological functions such as reproductive system development, membrane trafficking, embryo development ending in birth or egg hatching and regulation of growth. The two differentially expressed piRNAs (piR-28478 and piR-1077) showed alignment to protein coding gene (*PID1*) and processed pseudogene (RP11-1315F8), respectively. The piRNAs were also predicted to target 470 unique genes (Supplementary Table S4) with enrichment in ontology terms such as gliogenesis, membrane trafficking, vesicle-mediated transport, synaptic signaling, telencephalon and neuron projection development (Figure 1C).

Finally, we were interested to examine the overlap of differentially expressed spEV derived circRNAs identified in the present study with those identified in our previous study among men with poor, compared to normal, semen quality (Oluwayiose et al., 2022). Out of the 12 and 34 spEV circRNAs identified in the current and previous study, respectively, four (circ-0103367, circ-0009684, circ-0114168, circ-0001488) overlapped between the two studies (Supplementary Table S5). Of these, two (circ-0103367, circ-0009684) showed a consistent downregulation pattern and targeted genes that were enriched in important ontology terms including mitotic cell cycle, blood vessel morphogenesis, brain development, vesicle-mediated transport and growth (Supplementary Table S1).

## Discussion

To our knowledge, our study is the first to investigate the potential role of spEV-derived ncRNA and the subsequent ART outcome of successful live birth. In doing so, we leveraged our prospective cohort study, SEEDS, which recruited couples prior to undergoing infertility treatment and who were followed through their ART cycle that resulted with or without a live birth. Data for spEV ncRNA expression were obtained from banked seminal plasma samples from the same semen sample used for ART treatment. Sequencing of ncRNA libraries identified 12 spEV ncRNAs (10 circRNAs and 2 piRNAs) that were differentially expressed (q < 0.05) between live birth groups, in which predominantly (66.7%) were downregulated among men of couples without successful live birth. Finally, gene targets of the identified circRNAs and piRNAs were enriched in functional terms related to cellular communications, signaling, organ morphogenesis, and early embryonic development and birth.

Infertility treatment for couples has become prevalent especially in high income countries. However, the success of a live birth from one ART cycle has not improved over decades and results in multiple attempts that are accompanied by significant financial and emotional costs (Farley Ordovensky Staniec and Webb, 2007; Smith et al., 2015; Gleicher et al., 2019). In attempts to understand significant factors associated with pregnancy success prior to conception, the majority of previous studies have utilized machine learning algorithms to predict live birth outcomes based only on female characteristics (Metello et al., 2019; Liu et al., 2021). However, this resulted in low prediction accuracies as it failed to account for other effective predictors (Liu et al., 2021) like male factors, such as sperm DNA integrity (Nicopoullos et al., 2019), which has been reported to play critical role in embryo quality (Colaco and Sakkas, 2018). For example, our group has observed that, irrespective of female measurements, male chronological age (Oluwayiose et al., 2021) and preconception exposure to environmental phthalates (Wu et al., 2017a) were negatively associated with ART outcomes including fertilization, embryo quality and live birth. These associations were mediated by sperm DNA methylation at genomic loci related to embryo development (Wu et al., 2017b; Oluwayiose et al., 2021) and highlights the importance of male sperm epigenome in ART outcomes.

Other epigenetic mechanisms, such as EV-derived ncRNAs, are implicated in remodeling sperm RNAs during epididymal maturation and ejaculation (Du et al., 2016; Sharma et al., 2016; Jodar, 2019) and have also been observed to influence embryo development in mice and human (Chen et al., 2016; Sharma et al., 2018; Chen et al., 2021). In the present study, we found the expression of 12 spEVs ncRNAs were significantly altered among men of couples without live birth. Interestingly, four of the ten circRNAs identified in this study were also found to be differentially expressed in men with poor semen quality (Oluwayiose et al., 2022), in which two (circ-0103367, circ-0009684) showed a consistent downregulation pattern among men with poor semen quality and those without a successful live birth. The networks generated in enrichment analyses for the predicted gene targets of these two circRNAs revealed several functions related to signaling, vesicular transport, growth and development. This suggests spEV circRNAs may serve as important markers of both poor semen quality and couple reproductive outcomes.

circRNAs are single-stranded RNAs produced from alternative backsplicing events of corresponding messenger RNA (mRNA) resulting in a covalently closed-loop structures without 5′caps and 3′poly tails (Quan and Li, 2018). Despite the unique features of circRNAs, which include resistance to exonucleases, evolutionary conservation, high abundance, and participation in competing endogenous RNA network system, few studies have explored their roles in reproductive outcomes (Quan and Li, 2018). Advanced sequencing technologies have identified diverse species of circRNAs at different stages of pre-implantation embryos of human (Dang et al., 2016) and animal models (Fan et al., 2015; Kuang et al., 2020) with ontology terms related to organ and tissue morphogenesis, an observation consistent with our current findings.

The biological role of EV piRNAs in human body fluids in disease outcomes has been well documented (Goh et al., 2022). However, little is known about the contribution of EV piRNAs from seminal plasma with respect to reproductive outcomes. In the present study, two spEV piRNAs (piR-28478, piR-1077) were upregulated in men without live birth and predicted to regulate genes that are important in signaling, vesicle-mediated transport and neuron development. These piRNAs were also aligned within the protein coding region of *PID1* and the pseudogene, RP11-1315F8, respectively. PID1, also known as *NYGGF4*, encodes for a protein that contains a phosphotyrosine-interacting domain containing 1 and plays a role in diverse physiological processes including mitochondrion morphogenesis, signal transduction and glucose uptake (Zhao et al., 2010; Wu et al., 2011). The function of *PID1* has been suggested to differ based on cellular context, and its silencing in cell lines resulted in reduced proliferation rate (Monteleone et al., 2016). While the biological role of these two spEV piRNAs in relation to live birth is unknown and warrants further investigation, our observations suggest altered piRNAs in spEVs of men without live birth may be important in early development.

This study has notable strengths. We were the first to utilize high throughput small RNA sequencing to identify spEV ncRNAs that distinguished couples undergoing ART treatment that resulted with and without live birth. Additionally, the seminal plasma utilized in our study was collected from the same semen sample used for IVF treatment; thus, our prospective study design allowed us to connect spEV RNA cargo obtained during fertility treatment with subsequent live birth outcomes. We also acknowledge that this study has several limitations. First, spEV ncRNAs were compared between men of couples seeking infertility treatment; thus selection bias and the generalizability of our findings should be noted. Also, given there were no differences in couple demographic and male infertility status between the two live birth groups, we did not include these covariates in our statistical models. However, we cannot rule out the potential for other unmeasured confounders such as differences in nutrition, environmental exposure patterns or underlying health conditions. While the adaptor-ligation free protocol used for generating our small RNA libraries exhibits less sensitivity to modifications to RNA nucleotides resulting in more diverse libraries (Dard-Dascot et al., 2018), distinguishing naturally occurring and library-generated adenine bases at the 3’ end of libraries is a shortcoming. Lastly, our study did not investigate potential mechanisms by which spEV ncRNAs impact live birth outcomes. While our results suggest that select spEV ncRNAs provide prognostic value for predicting successful births, future studies should also focus on their association with early outcomes such as fertilization rates and embryonic development.

In conclusion, through the use of small RNA-sequencing, our study is the first, to our knowledge, to identify spEV ncRNA cargo that distinguished men of couples with and without successful live birth during infertility treatment. These findings highlight that spEVs, derived from several accessory sex glands, contain important molecular cargo that may impact the trajectory of early-life development and subsequent reproductive success, and moreover, the importance of the male partner for reproductive success.

## Data Availability

The datasets presented in this study can be found in online repositories. The names of the repository/repositories and accession number(s) can be found in the article/Supplementary Material.
